# Predator exposure early in life shapes behavioral development and individual variation in a clonal fish

**DOI:** 10.1038/s41598-024-72550-5

**Published:** 2024-09-17

**Authors:** U. Scherer, K. L. Laskowski, M. M. Kressler, S. M. Ehlman, M. Wolf, D. Bierbach

**Affiliations:** 1 SCIoI Excellence Cluster, Technische Universität Berlin, 10587 Berlin, Germany; 2 Faculty of Life Sciences, Humboldt-Universität Zu Berlin, 10117 Berlin, Germany; 3 Department of Fish Biology, Fisheries, and Aquaculture, Leibniz Institute of Freshwater Ecology and Inland Fisheries, 12587 Berlin, Germany; 4 Department of Evolution and Ecology, University of California Davis, Davis, CA 95616 USA; 5 Centre for Ecology and Conservation, University of Exeter, Penryn, Cornwall, TR10 9FE UK

**Keywords:** Ecology, Behavioural ecology, Freshwater ecology

## Abstract

Predation risk is one of the most important factors generating behavioral differences among populations. In addition, recent attention focusses on predation as a potential driver of patterns of individual behavioral variation within prey populations. Previous studies provide mixed results, reporting either increased or decreased among-individual variation in response to risk. Here, we take an explicit developmental approach to documenting how among-individual variation develops over time in response to predator exposure, controlling for both genetic and experiential differences among individuals. We reared juveniles of naturally clonal Amazon mollies, *Poecilia formosa*, either with or without a predator visible during feedings over 4 weeks and analyzed activity during feedings, time spent feeding and number of visits to the feeding spot. (I) Predator-exposed fish did not differ from control fish in average feeding behavior, but they were less active during feeding trials. (II) In the absence of the predator, substantial changes in among-individual variation over time were detected: among-individual differences in feeding duration increased whereas differences in activity decreased, but there were no changes in feeder visits. In contrast, in the presence of a predator, among-individual variation in all three behaviors was stable over time and often lower compared to control conditions. Our work suggests that predation risk may have an overall stabilizing effect on the development of individual variation and that differences in predation risk may well lead to population-wide differences in among-individual behavioral variation.

## Introduction

Predation risk is one of the major forces of natural selection shaping virtually every aspect of prey behavior, including, for example, grouping, activity, collective decision-making, mating, and foraging^1–7^. Many classic studies have focused on how predation risk influences average behavior at the population level. For example, poeciliid fish from high predation sites are on average bolder, more explorative, and more active than their conspecifics from low-predation sites^8–11^. And, with increasing predation risk, reef fish (several species, including parrotfish, *Sparisoma aurofrenatum* and surgeonfish, *Acanthurus bahianus*) consume drastically less food but fed at a faster rate^12^.

More recently, studies looking at consistent among-individual behavioral variation (aka individuality or animal personality), have highlighted that not all individuals within a population respond to a stimuli or cue in the same way^13–19^. This means that individuals consistently differ in their response to predation risk, producing patterns of consistent among-individual variation^20–24^ and co-variation among behaviors^25–28^. For example, when perceived predation risk was highest, Western mosquitofish, *Gambusia affinis*, exhibited the greatest consistent among-individual behavioral variation^22^. Similarly, wild-caught mud crabs, *Panopeus herbstii*, expressed more pronounced consistent among-individual variation in refuge use when presented with predator cues compared to a control condition were no such cues were presented^21^. Predation risk has thus been implicated as an important factor driving the emergence and maintenance of consistent individual behavioral variation^29^. However, not all studies found predation risk to lead to greater consistent among-individual variation in behavior within populations; several studies report a reverse pattern^30,31^. For example, wild-caught guppies, *Poecilia reticulata*, have been shown to exhibit reduced among-individual variation in their risk-taking behavior when presented with a computer-animated predator^30^.

This discrepancy in results (higher vs. lower degree of consistent among-individual behavioral variation in response to risk) may be rooted in several contributing factors. First, and perhaps most importantly, individual experience can play a vital role. That means, among-individual differences in the timeline, duration, intensity, or number of exposures can determine an individual’s risk-perception and, thus, its reaction towards predator cues^32–34^. In particular, exposure to predator cues early in life can have substantial and long-lasting effects^35–37^. Predicting such experiential effects may not be straightforward, for example, repeated exposures to threats can have diverse influences on behavior, alternatively leading to habituation or sensitization to the threat^34^. Second, populations can have diverse evolutionary histories (predator absence vs. predator presence, or spatial–temporal fluctuations in predation risk) leading to differences in genetic backgrounds (heterogenic populations where different genotypes may or may not react differently to predation risk vs. homogenic populations where individuals may behave more uniformly)^29,38,39^. Therefore, for a comprehensive understanding of how predation risk shapes behavioral variation at the individual level, we need studies that carefully control for both experiential and evolutionary backgrounds. Specifically, studies that take an explicit developmental approach to documenting components of behavioral variation in response to predation risk over time can foster our understanding of how and when predation risk generates patterns of behavioral divergence versus convergence^40–42^.

In the current study, we used a controlled experimental set-up to isolate the effects of predator exposure early in life on the development of individual behavioral variation. We do so by leveraging the naturally clonal Amazon molly, *P. formosa*. This clonal fish allows for the unique opportunity to genetically standardize test individuals and therefore isolate the effects of salient ecological experiences on patterns of individual behavioral development^43^. Two weeks after birth, juveniles were separated into virtually identical experimental environments. For the following 4 weeks, individuals were either reared in a predator treatment, where they were exposed to a natural piscine predator while foraging (i.e., visual contact with a convict cichlid during feedings), or a control treatment, where there was no predator present while foraging. In both treatments, we recorded individual feeding behavior (time spent feeding and number of visits to the feeding spot) and activity (average swimming speed) during periods where food was available; allowing us to estimate patterns of both within- and among-individual behavioral variation and hence estimate each behavior’s repeatability. Repeatability is a common metric used to quantify the degree of individuality within a population; it describes the extent to which differences among individuals explain the overall observed variation (including both among- and within-individual variation) in a given trait^18,44,45^. Amazon mollies are known to show strong individuality in both activity and feeding behavior when reared under benign control conditions^46–48^.

We predicted that (I) the predator exposure would influence average behavior at a population level, with fish in the predator treatment avoiding the predator, and therefore spending less time feeding, visiting the feeding spot less frequently, and being less active, compared to the control group. We further predicted that (II) individual behavioral variation in the three observed behaviors (time spent feeding, visits to the feeding spot, activity) would be altered by the predator exposure. Given that the predator may induce a trade-off between the relative risk of approaching and the reward of foraging, we predicted that fish in the predator treatment develop greater among-individual differences in behavior if they resolve this trade-off differently, leading to divergence (e.g., because individuals differ in their perception of risk or vulnerability)^32,33^. Alternatively, given the salience of predation risk on small schooling fish and the lack of genetic variation among our fish, we could instead expect individuals to conform on a behavioral response: avoid the predator or learn to ignore it^30^.

## Results

### (I) Reduced activity in the presence of the predator but no effect on feeding behavior

**Fig. 1:**
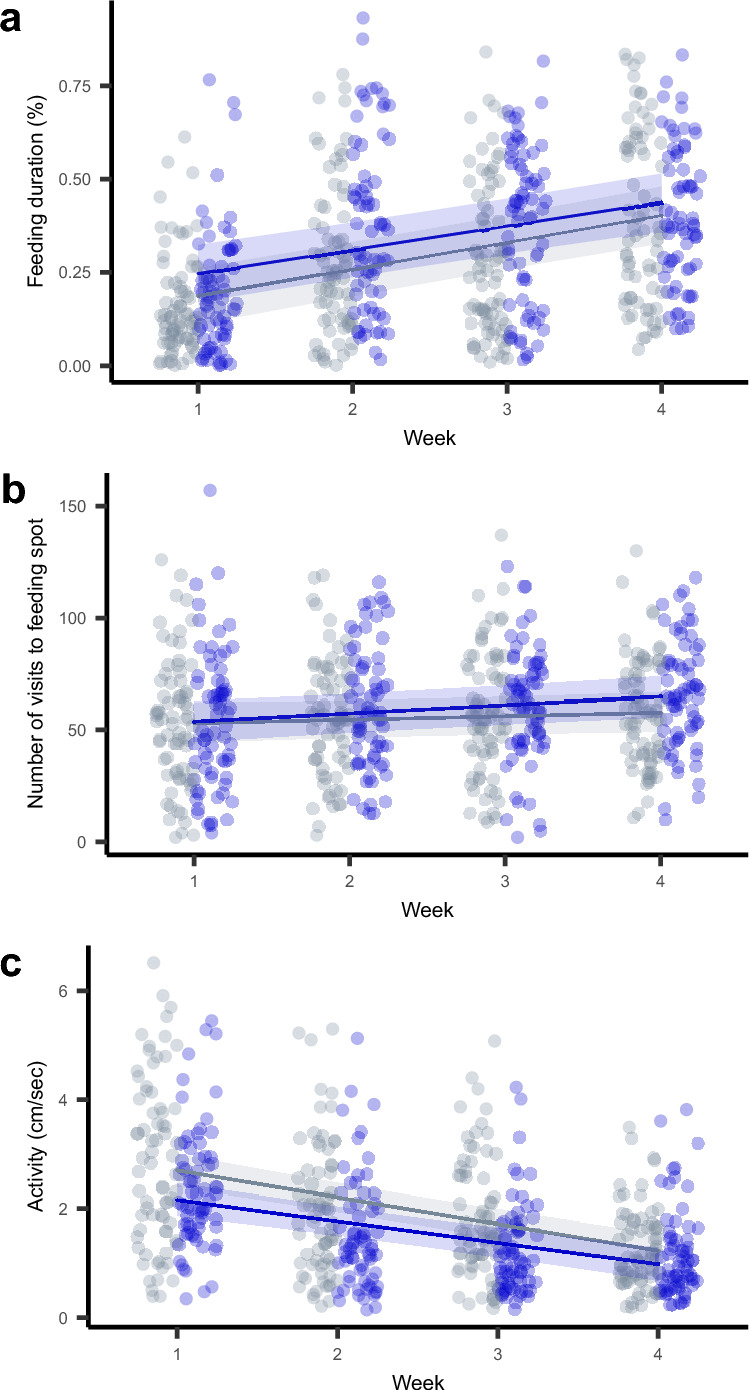
Effects of predator exposure on average behavior over time. (**a**–**c**) Presented are raw data (points, jittered horizontally for illustration purposes) and regression lines (gray = predator absence, blue = predator presence) with 95% confidence intervals, estimated from models including a non-significant treatment-week interaction term. (**a**, **b**) In both treatments, individuals increased their feeding duration and the number of visits to the feeding spot over time, but no difference between treatments was detected. (**c**) In the presence of the predator, individuals were on average less active than in the control, and in both treatments, individuals became less active over time.

We found neither a difference in feeding duration (Fig. 1a) nor in the number of visits to the feeding spot (Fig. 1b) between the predator and control treatment (Supplementary Table S1). And in both treatments, fish showed similar changes in feeding behavior over time (i.e., there was no evidence of a treatment x week interaction, Supplementary Table S1): fish significantly increased their time spent feeding (χ^2^ = 111.47, df = 1, *p* < 0.001; Supplementary Table S1, Fig. 1a), and visited the feeding spot significantly more often (χ^2^ = 11.14, df = 1, *p* = 0.001; Supplementary Table S1, Fig. 1b) as they got older. Fish in the predator treatment were generally less active than fish in the control treatment (χ^2^ = 4.305, df = 1, *p* = 0.038; Supplementary Table S1, Fig. 1c). There were no differences in how fish in the two treatments changed their activity over time (i.e., no treatment x week interaction, Supplementary Table S1) but rather individuals in both treatments decreased their activity over the course of the experiment (χ^2^ = 135.17, df = 1, *p* < 0.001; Supplementary Table S1, Fig. 1c). For all three behaviors, the main effects of treatment and week explained a modest amount of variation (marginal R^2^ < 0.19 for all three behaviors; Supplementary Table S1).

### (II) Stable and low among-individual variation in response to predator exposure

**Fig. 2:**
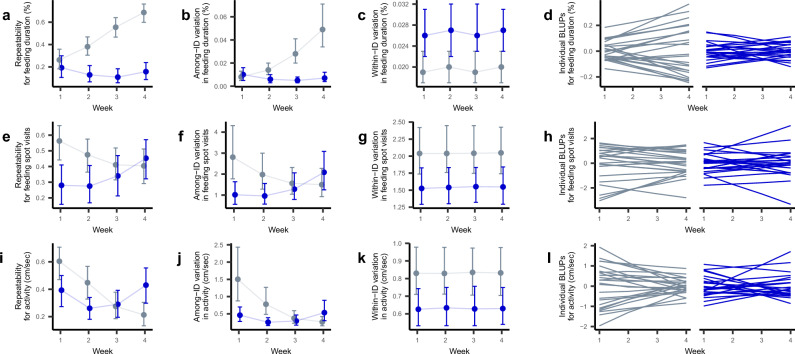
Effects of predator exposure on variance components. Shown are (**a**, **e**, **i**) repeatabilities, (**b**, **f**, **j**) among-individual variation, (**c**, **g**, **k**) within-individual variation, and (**d**, **h**, **l**) individual BLUPs (best linear unbiased predictors, i.e., random intercepts for individuals) for individuals in the predator (blue) vs. control (gray) treatment.

In general, we found that fish reared under predator exposure exhibited comparatively lower magnitudes of among-individual variation, and these levels of individual variation were very stable over time. In contrast, fish reared in the absence of the predator showed greater changes in the magnitude of among-individual variation over the 4-week experiment. The exact patterns of individual behavioral variation in our three different behaviors (feeding duration, visits to feeding spot, activity) differed from each other, both across the two treatment conditions and over time (Fig. 2).

For time spent feeding, we found that repeatability significantly increased over the course of the experiment when the predator was absent (i.e., in the control treatment) (Supplementary Table S2, Fig. 2a). The pattern was mainly driven by increases in among-individual variation, as within-individual behavioral variation remained stable throughout the course of the experiment (Supplementary Table S2, Fig. 2b,c). This resulted in a fanning-out pattern of individual BLUPs (best linear unbiased predictors, i.e., random intercepts for individuals) over the 4 experimental weeks (Fig. 2d). In contrast, in the predator treatment, both among-individual variation and repeatability remained stable and significantly lower than in the control treatment (Fig. 2a,b, Supplementary Table S2).

For the two other behaviors, number of visits to the feeding spot and activity, we found similar differences in the patterns of among-individual behavioral variation over time between the control and predators treatments, in that, among-individual variation was initially higher in the control treatment fish compared to the predator-exposed fish and decreased over the course of the experiment (the latter is statistically significant for activity only) (Fig. 2f and j; Supplementary Tables S3-S4), consequently, we observed a fanning-in pattern of individual BLUPs for activity in the control fish (Fig. 2l). In contrast, predator treatment fish exhibited more stable patterns of among-individual variation over the 4 weeks (Supplementary Tables S3-S4, Fig. 2e-l). For both behaviors, within-individual variation remained stable over time and similar in both treatments (Supplementary Tables S3-S4, Fig. 2g and k).

Interestingly, we found significant brood differences in feeding behavior (time spent feeding as well as number of visits to the feeder) among predator-exposed fish; but not among control-fish (Supplementary Tables S2-S3). Conversely, we found repeatable brood differences in activity among control- but not predator-fish, although, here, the repeatabilities are negligibly low, i.e., these repeatabilities explained only approx. 1–2% of the total variation in activity (Supplementary Tables S4).

## Discussion

We tested how exposure to a natural predator early in life may alter the development of consistent among-individual behavioral variation. To do so, we used a clonal fish, the Amazon molly, allowing us to isolate the effects of this salient experience on behavioral development, while controlling for genetic variation^43^. We found that (I) fish exposed to predators were on average less active than fish reared under control conditions, but there was no change in average feeding behavior (time spent feeding and visits to feeding spot) in response to the predator. (II) In the control, among-individual variation changed substantially over time, with contrasting patterns depending on the behavior of interest: we observed a strong divergence, i.e., increase in among-individual variation, in the time fish spent feeding, and a convergence, i.e., decrease in among-individual variation, in activity; but no changes in feeder visits. Predator-exposed fish, on the other hand, showed no signs of change in among-individual variation over time and showed an overall tendency to behave more similarly to each other compared to control fish.

In line with much previous work, individuals were on average less active in the presence of the predator^6,49^, though there was no decrease in their average feeding behavior (feeding duration and number of visits to the feeding spot) compared to control individuals. The predator’s lack of influence on feeding behavior may be explained by the predation risk allocation hypothesis, which postulates that as exposure to predation risk increases, individuals reduce their avoidance of predators because the associated loss of energy intake becomes too significant^50^. Our fish showing predator avoidance by reducing their activity may indicate that the two behaviors differ in their cost–benefit trade-offs. That is, decreasing both activity and feeding can help minimize overall exposure to danger^51–53^, however, feeding is crucial for energy intake and cannot be entirely avoided, whereas reducing activity may be less costly.

The presence vs. absence of the predator had profound consequences on the development of behavioral variation in our Amazon mollies with two general trends. First, predator-exposed fish exhibited generally lower among-individual variation compared to fish in the predator-free control scenario, indicating that individuals may agree in their perception of the predator as a potential threat and align in their risk assessment, leading to individuals conforming to a behavioral strategy that might reflect an ‘optimal response’^30,54^. Second, while we observed substantial changes in among-individual variation over time in control fish (see below for developmental processes potentially contributing to the pattern observed), among-individual variation among predator-exposed fish remained stable. The lack of change suggests that individual decisions about how to behave in the presence of the predator were persistent. These results likely indicate that the predator was perceived as a potential threat throughout the experimental period. Previous studies demonstrate innate predator recognition of predator-naïve poeciliid fishes when confronted with piscivorous and omnivorous cichlids^3,55^ (see^56–60^ for further studies demonstrating innate predator recognition in fishes). Thus, it is highly likely that behavioral responses of Amazon mollies to the cichlids used in the present study, which are a natural predator for mollies, represent anti-predator responses to perceived predation risk and not just general fear responses towards a new stimulus in our test fish.

Individuals aligning their behavior under potential risk could have significant ecological consequences. In particular, the suppression of the development of among-individual diversity in feeding behavior in response to predator exposure might lead to reduced differences in growth and body size among individuals. This, in turn, could impact a wide range of fitness-relevant intraspecific interactions that are dependent on body size, including competition and hierarchies^61,62^, and reproductive behaviors^63^. Additionally, it may affect interspecific interactions, such as prey oddity and consequently predator hunting success^64^. Reduced variation in activity among individuals may impair processes related to the acquisition of environmental information and therefore adaptation if individuals behaving more alike translates into individuals acquiring similar or fewer information. While we can here only speculate about the consequences of predator-induced homogenization of prey behavior, future studies may address these questions more specifically.

Our finding of behavioral alignment under risk contrasts with several other studies indicating that predation risk promotes among-individual variation^20–23^. In our study, we strongly reduced variation in both genetic and experiential background by testing genetically identical individuals with no prior predator exposure in a highly standardized procedure. We thereby demonstrate that genes and/or experiential differences may drive the development of among-individual variation under risk as observed elsewhere^20–23^. Future experimental investigations could build upon our results by manipulating either of these two factors independently (e.g., by using the sexually reproducing parental species of the Amazon molly or different clonal lines), allowing for a more detailed examination of the distinct roles played by genes and experience.

We observed strong among-individual diversification in the time spent feeding when the predator was absent (while no such development was observed in the presence of the predator). Potentially, this pattern could be caused by individuals differing in their perception of whether the feeding apparatus (bottle with feeder) introduced into the tank during feeding trials posed a threat. That means, under control conditions, there might have been higher environmental uncertainty (not clear if the bottle is dangerous) compared to the predator treatment (where the predator itself represents an accurate cue about potential risk) leading to more pronounced differences in how individuals perceive and assess risk^65,66^. Initial differences in risk assessment may be enhanced via positive behavior-state feedback loops leading to pattern of behavioral divergence over time^41,67^. This way, even small initial differences in the perception of potential risk could become substantial over time. Future studies may investigate the role of environmental uncertainty and feedback loops in the development on among-individual behavioral variation in more detail.

Regarding activity, we observed high among-individual variation under control conditions to begin with, which then strongly decreased over the experimental period. This pattern of behavioral convergence may potentially be a by-product of individuals becoming on average less active (fewer differences among individuals are possible when the total behavioral range gets smaller), which in turn may reflect a response to the environment, specifically adaptation or habituation. Individual environments were stable and simple throughout the experiment and while being active is energetically costly the expected benefits of exploring this specific experimental environment were rather low. Declining activity could potentially also be caused by illness, however, all fish appeared healthy during trials as well as during the weeks following our experiment.

In conclusion, we observed that predator-exposed fish were on average less active but did not reduce feeding behavior compared to control fish. These population-level responses towards the predator indicate that individuals make strategic decisions about how to avoid a potential threat depending on the specific trade-offs (i.e., no predator avoidance when avoidance is too costly), thereby supporting the risk allocation hypothesis. Importantly, the presence of the predator led to behavioral conformance (potentially reflecting an optimal response) characterized by lower variation among individuals, which remained stable over the course of the experiment, compared to higher and more variable among-individual differences under control conditions. This suggests that when confronted with a predator, individuals align their risk evaluation, whereas in the absence of an immediate threat, variation in environmental perception among individuals may become more pronounced. Thus, next to the effects predators have on average population level behavior, there are also important—and largely overlooked—effects on the development of variation among individuals, with potentially important ecological and evolutionary consequences.

## Methods

### Study species and animal care

The Amazon molly is a gynogenetically reproducing freshwater fish from the subtropics of North America^68–72^ and the first discovered clonal vertebrate^73,74^, stemming from a single hybridization event between a female Atlantic molly, *Poecilia mexicana*, and a male sailfin molly, *Poecilia latipinna*, about 100,000 years ago^71,72^; but see^75^ for a population genomics study suggesting a history of backcrossing with the parental species before the onset of gynogenesis. Gynogenetic reproduction means that Amazon mollies require sperm from one of their parental species to trigger embryonic development, but paternal genetic material is not incorporated into the egg^69,71,72^ except in rare cases of male DNA fragment introgression^76–78^. Resulting offspring are therefore genetically identical to their mother and each other (except minute genetic differences created, e.g., by mutation).

Our experimental fish were lab-reared descendants of wild-caught fish originating from waters around the Mexican city of Tampico. Regular molecular checks confirmed that all *P. formosa* individuals used were clones^79^. Fish were maintained in large, uni-clonal tanks (200 L) with several *P. mexicana* males as sperm donors and were fed twice a day (7 days a week) with flake food (TetraMin) and once a week with live or frozen *Chironomid* larvae. Temperature was maintained at 25 °C with a 12/12 light/dark circle provided.

## Experimental procedure

**Fig. 3:**
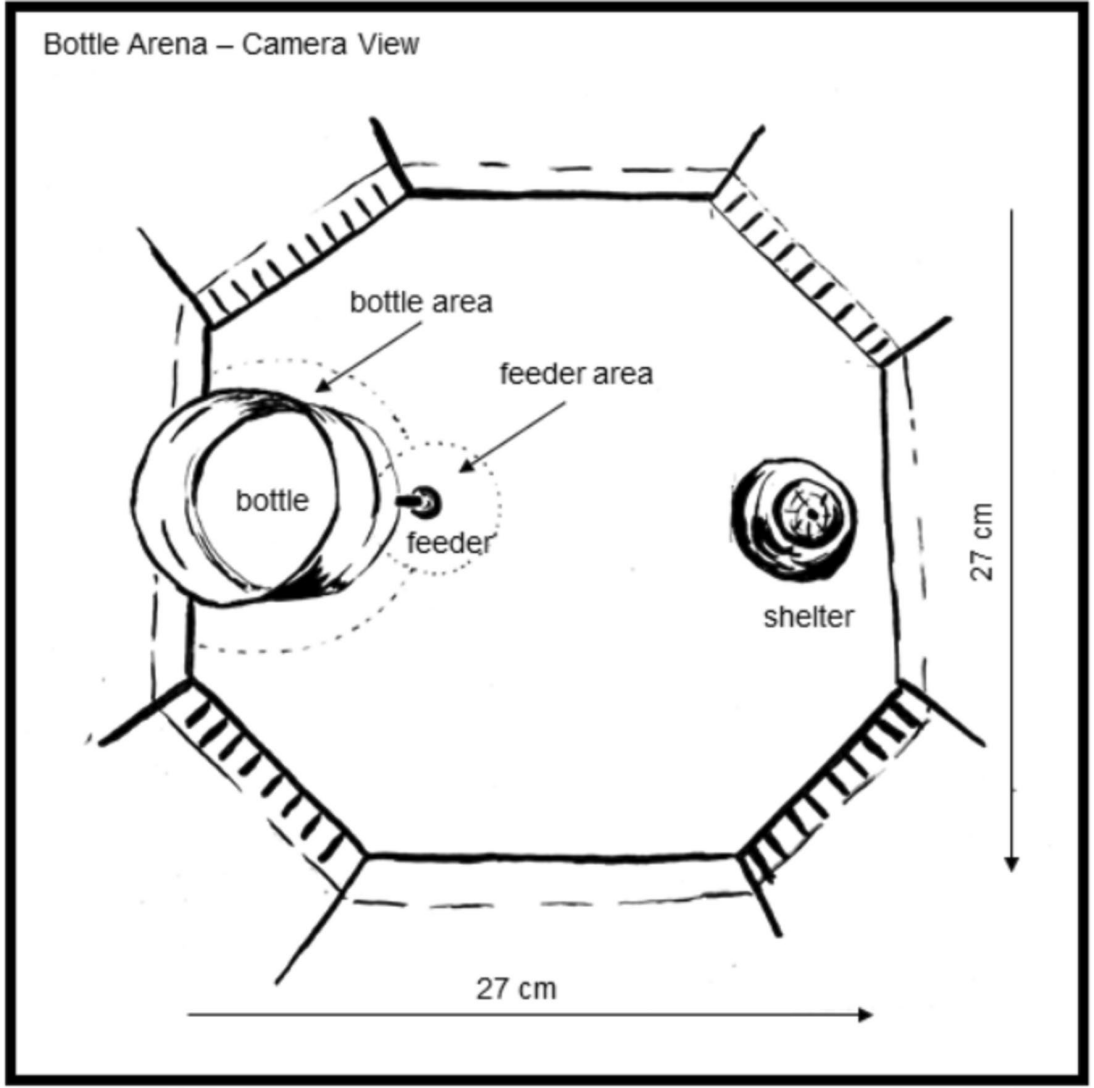
Experimental set-up of feeding trials. Illustrated is a single experimental tank (tank size: 27 cm × 27 cm, corners (banded) serve as water in- and outflow to maintain water quality and connect the tank to the filter system) with a permanent shelter (up-side-down flower pot, 4.5 cm lower diameter). The bottle (without predator, bottle: 7.6 cm diameter, bottle area: 11.2 cm), including the feeder (food tablet (Tetra WaferMix) and clamp, feeder area: 4.5 cm diameter), was introduced at a standardized position into the tank during feeding trials.

Before this study, we isolated several gravid females from one clonal line into smaller tanks (5 L) equipped with artificial plants and the same water conditions and feeding regime as outlined above. After parturition, we immediately removed the mothers (*N* = 6) and raised their broods for 2 weeks on *Artemia nauplii* and dusted flake food (TetraMin). We then placed offspring individually in one of 24 experimental tanks (Fig. 3), which we used in succession as broods were born at different times. We employed a split-brood design, i.e., for each brood, half of the individuals were allocated to the control treatment and the other half were allocated to the predator treatment (in total, *N* (control) = 24 fish and *N* (predator) = 23 fish). After 2 days of habituation during which the feeding of the first 2 weeks was continued, we started our experimental feeding trials: fish in the control treatment were presented with a water-filled bottle at which we clamped a food tablet (Fig. 3). Fish in the predator treatment were presented with the same bottle and food tablet but this time, the bottle held a convict cichlid (*Amatitlania nigrofasciata,* total length: 3.96 cm ± 0.62 cm (mean ± SD)). Predators were allowed to acclimate for 3–5 min inside the bottle, before the bottle was added to the experimental tank. For each trial, predators were chosen randomly from a pool of 100 juvenile cichlids (200 L tank, holding conditions as above). Bottles were put into the tanks every Monday, Wednesday, and Friday for 4 weeks so that each molly was presented 12 times with either an empty or a predator bottle. After the bottle with the food tablet was inserted, we gave our fish 30 min to feed and recorded at 2 frames per second from above^47^. Test individuals were not habituated to the feeder prior to starting experimental feeding trials. On days without feeding trials (i.e. Tuesdays, Thursdays, and Sundays; no feeding on Saturdays), fish were fed in the morning with a food tablet and the remaining food was removed after 3 h. Videos from the feeding trials were tracked using software Ethovision XT (Noldus Inc.). We assessed the time spent feeding as time spent in the feeder area (‘feeding duration’, in %), how many times the fish visited the feeder area (‘number of visits to feeding area’) and average velocity (‘activity’, in cm/sec) for each trial (Fig. 3). In addition to these behavioral variables, we used the detected blob sizes during the tracking as approximation of the fish’s body sizes at the first day of testing (day 1) and at the last day (day 12). Individuals allocated to the two treatments did not differ in body size from each other; either at the beginning or at the end of feeding trials (see Supplementary Figure S1 and Supplementary Table S6 for more details). The experimental set-up, including camera and feeding cylinder position was optimized to capture all facets of the molly behavior, which, however, circumvented the analysis of the cichlids’ behavior. Nevertheless, we observed cichlids swimming regularly in their cylinders during the trials (pers. observation).

The predator fish used in this experiment, the convict cichlid, is a natural predator for poeciliid fishes stemming from central America and is introduced worldwide due to releases from the ornamental trade^80^. Previous studies on closely related poeciliid fishes (*P. reticulata* and *P. mexicana*) demonstrate that predator-naïve individuals show typical anti-predator responses (altered mating preferences and avoidance behavior) when presented with predatory and omnivorous cichlids (*Cichlasoma salvini, Crenicichla alta, Andinoacara pulcher, Petenia splendida*), indicating that the predators are perceived as such^3,30,55^. These studies further show that behavior expressed in the presence of these predators is distinctly different compared to behavior expressed in the presence of a related, non-predatory species (*Xiphophorus hellerii*)^3,55^. See, e.g.,^56–60^ for further studies demonstrating innate predator recognition in fishes.

### Statistical analyses

#### General details

Statistical analyses were performed in R 4.2.1^81^. LMMs (linear mixed-effect models) were fitted using the *lmer*-package^82^. Variance components (repeatabilities, among-, and within-individual variation) with 95% confidence intervals (CIs) were estimated from LMMs following^83^. We tested whether variance components differed significantly between treatments or weeks by comparing the 95% Cis of estimates (significant difference when CIs do not overlap). Significance for behavioral repeatabilities was derived from the 95% CIs being distinctly different to zero. Model assumptions were verified using q-q plots and residual plots. We included random intercepts for both individuals and broods in all models, however, in cases where brood ID had no explanatory power, the term was removed from the model. Complete model structures, including random terms, are provided as Supplementary Table S1 and S5.

Our three target variables (feeding duration, visits to feeding spot, activity) were not (feeding duration and visits to feeding spot), moderately (activity and feeding duration), or weakly (activity and visits to feeding spot) predictive of each other (for more information and R^2^ see Supplementary Table S5), we therefore did not consider these variables redundant.

### (I) Does predator exposure affect average behavior?

We tested if average behavior differed between the predator and control treatment by building LMMs with the behavior of interest as the response (time spent feeding, visits to the feeding spot, activity) and treatment (predator vs. control) as fixed effects. We further included week (1–4) as a continuous fixed effect to test if average behavior changed over development (e.g., fish getting older or habituating over the course of the experiment), as well as the week x treatment interaction term to test for potential differences between the two treatments in how behavior may have changed over time. The interaction term was removed from the models when non-significant. We included random intercepts for both individuals and broods (*N* (total, i.e., predator and control) = 559 observations from 47 individuals and 6 broods per model).

### (II) Does predator exposure affect individual behavioral variation?

To test if predator exposure affected behavioral variation, we estimated the among- and within-individual variation, and hence repeatability, for each of our three target behaviors (time spent feeding, visits to the feeding spot, activity) in the two treatments (predator vs. control) separately. That is, for each behavior and treatment, we ran an LMM with the behavior of interest as response and experimental week (1–4) as continuous fixed effect (in total 6 models, for each behavior: *N* (control) = 285 observations from 24 individuals and *N* (predator) = 274 observations from 23 individuals). As random terms, we included individual intercepts and individual slopes as well intercepts for brood. To assess how variance components may change over the course of the experiment, we ran each of the above 6 models 4 times, where we centered the model to a different week in each run. That is, we ‘sliced’ the model at different time points allowing us to estimate the variance components for each week (because random intercepts are estimated where fixed effects, in this case, ‘week’, are equal to 0)^47^.

## Supplementary Information

Supplementary Information.

## Data Availability

The data generated in this study are deposited in Figshare.
