# Supplementary material for: Predator exposure early in life shapes behavioral development and individual variation in a clonal fish
Source: Sci Rep. 2024 Sep 17;14:21668. doi: 10.1038/s41598-024-72550-5 (PMC11408663; doi:10.1038/s41598-024-72550-5)
Supplement: Supplementary file 1 — Supplementary Information. [file 41598_2024_72550_MOESM1_ESM.pdf]

SUPPLEMENTARY INFORMATION FOR:

Predator exposure early in life shapes behavioral development and individual variation in a clonal fish

Scherer U[1,2,3]*, Laskowski KL[3,4], Kressler MM[3,5], Ehlman SM[1,2,3], Wolf M[1,3], Bierbach D[1,2,3]

[1] SCIoI Excellence Cluster, Technische Universität Berlin, 10587 Berlin, Germany

[2] Faculty of Life Sciences, Humboldt-Universität zu Berlin, 10117 Berlin, Germany

[3] Department of Fish Biology, Fisheries, and Aquaculture, Leibniz Institute of Freshwater Ecology and Inland Fisheries, 12587 Berlin, Germany

[4]Department of Evolution & Ecology, University of California Davis, Davis CA 95616, USA

[5]Centre for Ecology and Conservation, University of Exeter, Penryn, Cornwall, TR10 9FE

*Corresponding author (u.k.scherer@gmail.com)

Table S1 Linear mixed-effects models testing if individuals that were forced to forage in the presence of the predator behaved differently over time (regarding their activity, time spent feeding, and visits to the feeding spot) compared individuals in the control treatment. We considered that, possibly, behaviors may develop differently over time in the two treatments by including an interaction term between week and treatment (full models, left), however, if not significant, the interaction term was removed from the models (final models, right). We included random intercepts for both individuals and broods in all models. In cases where brood had no explanatory power, the term was removed from the model.

		Full model					Final model				
Response	*Predictors*	*Estimate*	*SE*	χ^2	*p*	*df*	*Estimate*	*SE*	χ^2	*p*	*df*
Feeding duration (%)	(Intercept)	0.114	0.042	-	-	-	0.125	0.040	-	-	-
	Treatment [Predator]	0.069	0.044	-	-	-	0.047	0.031	2.224	0.136	1
	Week	0.072	0.009	-	-	-	0.068	0.006	111.470	<0.001	1
	Treatment [Predator] × Week	-0.009	0.012	0.519	0.472	1	-	-	-	-	-
	Random Effects										
	σ^2	0.03					0.03				
	τ_{00}	0.01 Individual					0.01 Individual				
		0.00 Brood					0.00 Brood				
	ICC	0.35					0.35				
	N	47 Individual					47 Individual				
		6 Brood					6 Brood				
	Observations	559					559				
	Marginal R^2 / Conditional R^2	0.138 / 0.438					0.138 / 0.437				
Visits to feeding spot	(Intercept)	51.82	4.967	-	-	-	49.268	4.602	-	-	-
	Treatment [Predator]	-1.41	6.404	-	-	-	3.797	5.141	0.542	0.462	1
	Week	1.55	1.073	-	-	-	2.578	0.768	11.143	0.001	1
	Treatment [Predator] × Week	2.09	1.534	1.858	0.173	1	-	-	-	-	-
	Random Effects										
	σ^2	408.60					410.10				
	τ_{00}	273.37 Individual					273.14 Individual				
		22.16 Brood					22.11 Brood				
	ICC	0.42					0.42				
	N	47 Individual					47 Individual				
		6 Brood					6 Brood				
	Observations	559					559				
	Marginal R^2 / Conditional R^2	0.018 / 0.430					0.017 / 0.428				
Activity (cm/sec)	(Intercept)	3.189	0.178	-	-	-	3.074	0.156	-	-	-
	Treatment [Predator]	-0.626	0.255	-	-	-	-0.391	0.184	4.305	0.038	1
	Week	-0.487	0.049	-	-	-	-0.440	0.035	135.17	<0.001	1
	Treatment [Predator] × Week	0.095	0.071	1.791	0.181	1	-	-	-	-	-
	Random Effects										
	σ^2	0.87					0.87				
	τ_{00}	0.32 Individual					0.32 Individual				
	ICC	0.27					0.27				
	N	47 Individual					47 Individual				
	Observations	559					559				
	Marginal R^2 / Conditional R^2	0.192 / 0.412					0.190 / 0.410				

Table S2 Variance components for time spent feeding in the control vs. predator treatment, over the 4 experimental weeks.

Behavior	Variance component	Week	Control			Predator		
			Estimate	Lower CI	Upper CI	Estimate	Lower CI	Upper CI
Feeding duration (%)	Repeatability for individual	1	0.263	0.183	0.359	0.193	0.106	0.300
	Repeatability for Brood		0.000	0.000	0.000	0.266	0.092	0.463
	Among-individual variation in intercepts		0.008	0.005	0.013	0.010	0.005	0.016
	Among-individual variation in slopes		0.003	0.002	0.005	0.001	0.001	0.002
	Among-brood variation in intercepts		0.000	0.000	0.000	0.014	0.004	0.032
	Within-individual variation		0.019	0.017	0.023	0.026	0.022	0.031
	Repeatability for individual	2	0.381	0.305	0.469	0.129	0.067	0.212
	Repeatability for Brood		0.000	0.000	0.000	0.255	0.098	0.454
	Among-individual variation in intercepts		0.014	0.010	0.020	0.006	0.003	0.010
	Among-individual variation in slopes		0.003	0.002	0.005	0.001	0.001	0.002
	Among- brood variation in intercepts		0.000	0.000	0.000	0.012	0.004	0.027
	Within-individual variation		0.020	0.017	0.023	0.027	0.022	0.032
	Repeatability for individual	3	0.555	0.467	0.641	0.109	0.059	0.184
	Repeatability for Brood		0.000	0.000	0.000	0.264	0.089	0.435
	Among-individual variation in intercepts		0.028	0.020	0.041	0.005	0.003	0.008
	Among-individual variation in slopes		0.003	0.002	0.004	0.001	0.001	0.002
	Among- brood variation in intercepts		0.000	0.000	0.000	0.012	0.003	0.026
	Within-individual variation		0.019	0.017	0.023	0.026	0.023	0.032
	Repeatability for individual	4	0.687	0.599	0.762	0.158	0.087	0.239
	Repeatability for Brood		0.000	0.000	0.000	0.246	0.096	0.409
	Among-individual variation in intercepts		0.049	0.034	0.071	0.007	0.004	0.012
	Among-individual variation in slopes		0.003	0.002	0.003	0.001	0.001	0.002
	Among- brood variation in intercepts		0.000	0.000	0.000	0.011	0.004	0.024
	Within-individual variation		0.020	0.017	0.023	0.027	0.023	0.031

Table S3 Variance components for the number of visits to the feeding spot in the control vs. predator treatment, over the 4 experimental weeks.

Behavior	Variance component	Week	Control			Predator		
			Estimate	Lower CI	Upper CI	Estimate	Lower CI	Upper CI
Visits to feeding spot	Repeatability for individual	1	0.563	0.442	0.661	0.280	0.159	0.410
	Repeatability for Brood		0.000	0.000	0.000	0.242	0.080	0.428
	Among-individual variation in intercepts		2.807	1.772	4.310	1.015	0.562	1.635
	Among-individual variation in slopes		0.134	0.084	0.209	0.186	0.114	0.296
	Among-brood variation in intercepts		0.000	0.000	0.000	0.878	0.250	1.968
	Within-individual variation		2.039	1.737	2.420	1.526	1.293	1.828
	Repeatability for individual	2	0.475	0.364	0.575	0.275	0.168	0.407
	Repeatability for Brood		0.000	0.000	0.000	0.223	0.085	0.408
	Among-individual variation in intercepts		1.977	1.284	2.998	0.959	0.572	1.557
	Among-individual variation in slopes		0.144	0.086	0.232	0.185	0.108	0.299
	Among-brood variation in intercepts		0.000	0.000	0.000	0.769	0.258	1.771
	Within-individual variation		2.039	1.758	2.448	1.540	1.301	1.832
	Repeatability for individual	3	0.411	0.315	0.518	0.340	0.212	0.470
	Repeatability for Brood		0.000	0.000	0.000	0.201	0.071	0.374
	Among-individual variation in intercepts		1.560	1.040	2.318	1.283	0.795	2.057
	Among-individual variation in slopes		0.159	0.094	0.269	0.177	0.108	0.279
	Among-brood variation in intercepts		0.000	0.000	0.000	0.748	0.227	1.753
	Within-individual variation		2.042	1.747	2.448	1.552	1.321	1.833
	Repeatability for individual	4	0.404	0.291	0.512	0.453	0.321	0.571
	Repeatability for Brood		0.000	0.000	0.000	0.168	0.061	0.319
	Among-individual variation in intercepts		1.497	0.926	2.271	2.086	1.252	3.082
	Among-individual variation in slopes		0.150	0.091	0.245	0.156	0.103	0.224
	Among-brood variation in intercepts		0.000	0.000	0.000	0.773	0.239	1.649
	Within-individual variation		2.048	1.741	2.425	1.548	1.294	1.841

Table S4 Variance components for activity in the control vs. predator treatment, over the 4 experimental weeks.

Behavior	Variance component	Week	Control			Predator		
			Estimate	Lower CI	Upper CI	Estimate	Lower CI	Upper CI
Activity (cm/sec)	Repeatability for individual	1	0.604	0.470	0.707	0.393	0.273	0.500
	Repeatability for Brood		0.021	0.005	0.059	0.000	0.000	0.000
	Among-individual variation in intercepts		1.503	0.873	2.433	0.460	0.277	0.699
	Among-individual variation in slopes		0.103	0.076	0.132	0.084	0.055	0.121
	Among-brood variation in intercepts		0.052	0.012	0.143	0.000	0.000	0.000
	Within-individual variation		0.830	0.710	0.978	0.626	0.532	0.743
	Repeatability for individual	2	0.448	0.328	0.567	0.261	0.181	0.340
	Repeatability for Brood		0.010	0.002	0.029	0.000	0.000	0.000
	Among-individual variation in intercepts		0.778	0.485	1.267	0.256	0.159	0.388
	Among-individual variation in slopes		0.108	0.073	0.156	0.093	0.056	0.140
	Among-brood variation in intercepts		0.018	0.004	0.053	0.000	0.000	0.000
	Within-individual variation		0.829	0.712	0.977	0.634	0.533	0.750
	Repeatability for individual	3	0.275	0.185	0.378	0.290	0.195	0.392
	Repeatability for Brood		0.013	0.003	0.036	0.000	0.000	0.000
	Among-individual variation in intercepts		0.371	0.226	0.591	0.292	0.170	0.467
	Among-individual variation in slopes		0.126	0.077	0.193	0.089	0.056	0.138
	Among-brood variation in intercepts		0.017	0.005	0.051	0.000	0.000	0.000
	Within-individual variation		0.835	0.706	0.973	0.628	0.533	0.756
	Repeatability for individual	4	0.213	0.133	0.304	0.430	0.298	0.555
	Repeatability for Brood		0.013	0.003	0.032	0.000	0.000	0.000
	Among-individual variation in intercepts		0.264	0.152	0.421	0.534	0.300	0.892
	Among-individual variation in slopes		0.127	0.071	0.204	0.077	0.053	0.112
	Among-brood variation in intercepts		0.016	0.004	0.042	0.000	0.000	0.000
	Within-individual variation		0.832	0.704	0.976	0.630	0.540	0.749

Table S5 Linear mixed-effects models testing if our three target behaviors (activity, feeding duration, visits to feeding spot) predicted each other: activity and feeding duration as well as activity and visits to the feeding spot were positively related to each other but feeding duration and number of visits to the feeding spot did not predict each other; tested for the predator (left models) and control (right models) treatment separately. We included random intercepts for both individuals and broods in all models, however, in cases where brood had no explanatory power, the term was removed from the model.

Response	Predictors	Predator					Control				
		Estimate	*SE*	χ^2	*p*	*df*	*Estimate*	*SE*	χ^2	*p*	*df*
Activity (cm/sec)	(Intercept)	2.779	0.121	-	-	-	3.208	0.163	-	-	-
	Feeding duration (%)	-3.508	0.235	162.530	<0.001	1	-4.301	0.286	164.190	<0.001	1
	**Random Effects**										
	σ^2	0.52					0.70				
	τ_{00}	0.15 Individual					0.42 Individual				
	ICC	0.22					0.37				
	N	23 Individual					24 Individual				
	Observations	274					285				
	Marginal R^2 / Conditional R^2	0.457 / 0.577					0.428 / 0.643				
Activity (cm/sec)	(Intercept)	0.982	0.204	-	-	-	1.133	0.201	-	-	-
	Visits to feeding spot	0.010	0.003	10.521	0.001	1	0.015	0.003	22.216	<0.001	1
	**Random Effects**										
	σ^2	0.94					1.25				
	τ_{00}	0.10 Individual					0.21 Individual				
		0.04 Brood									
	ICC	0.13					0.14				
	N	23 Individual					24 Individual				
		6 Brood									
	Observations	274					285				
	Marginal R^2 / Conditional R^2	0.063 / 0.189					0.098 / 0.227				
Feeding duration (%)	(Intercept)	0.329	0.054	-	-	-	0.269	0.044	-	-	-
	Visits to feeding spot	0.001	0.001	1.160	0.281	1	0.000	0.000	0.371	0.543	1
	**Random Effects**										
	σ^2	0.03					0.03				
	τ_{00}	0.00 Individual					0.01 Individual				
		0.01 Brood					0.00 Brood				
	ICC	0.28					0.33				
	N	23 Individual					24 Individual				
		6 Brood					6 Brood				
	Observations	274					285				
	Marginal R^2 / Conditional R^2	0.005 / 0.287					0.001 / 0.327				

Table S6 Linear mixed-effects models testing for body size differences between treatments and over time. We built a linear mixed model with approximated body sizes (detected blob size during tracking with Ethovision) as response and treatment (control vs. predator), trial number (trial 1 vs. trial 12), and the treatment-trial interaction term as fixed effects. Individual and brood ID were included as random terms. Body size approximations are smaller than actual fish sizes

		Full model					Final model				
Response	*Predictors*	*Estimate*	*SE*	χ^2	*p*	*df*	*Estimate*	*SE*	χ^2	*p*	*df*
Body size approximation (detected blob size during tracking, in cm)	(Intercept)	0.726	0.015	-	-	-	0.727	0.014	-	-	-
	Treatment [Predator]	0.024	0.014	-	-	-	0.021	0.012	3.072	0.081	1
	Trial [End]	0.102	0.009	-	-	-	0.099	0.007	87.624	<0.001	1
	Treatment [Predator] × Trial [End]	-0.005	0.013	0.120	0.729	1	-	-	-	-	-
	**Random Effects**										
	σ^2	0.00					0.00				
	τ_{00}	0.00 Individual					0.00 Individual				
		0.00 Brood					0.00 Brood				
	ICC	0.63					0.63				
	N	48 Individual					48 Individual				
		7 Brood					7 Brood				
	Observations	96					96				
	Marginal R^2 / Conditional R^2	0.477 / 0.809					0.477 / 0.808				

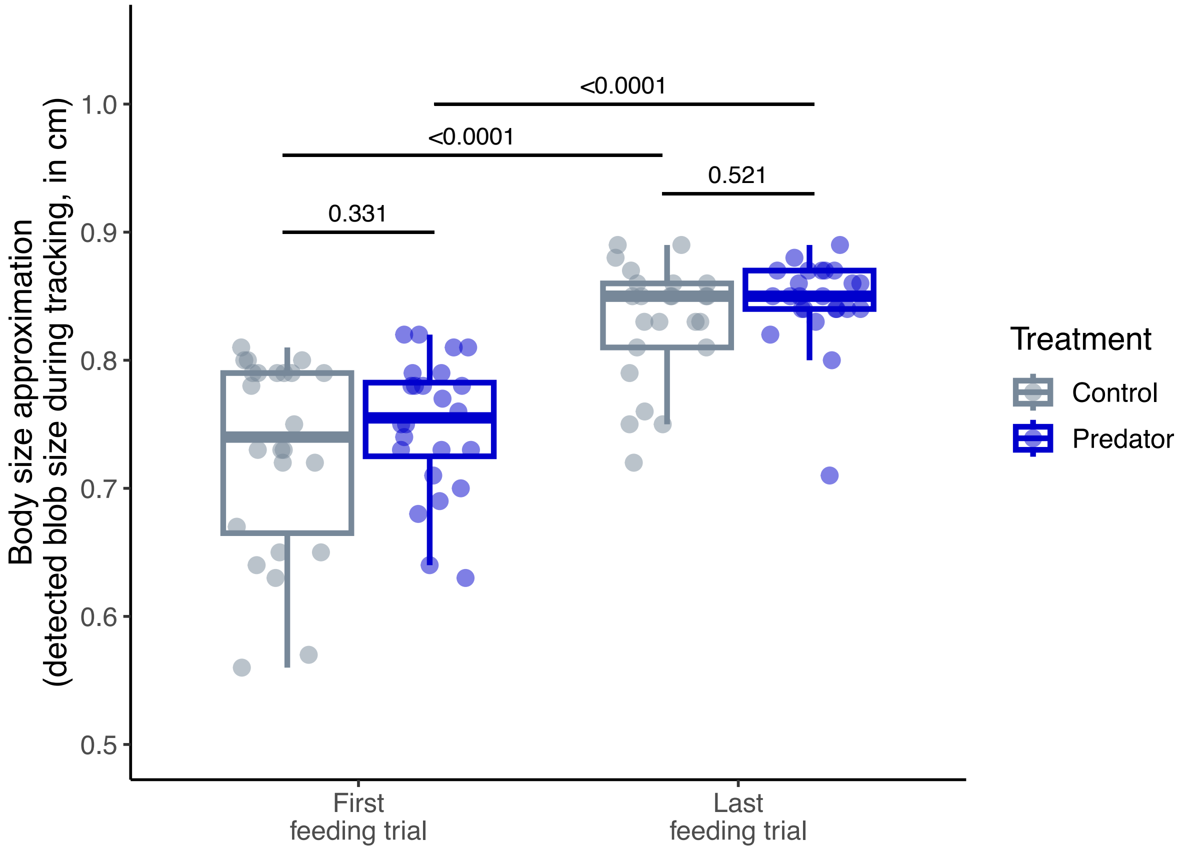

Figure S1. Body sizes of Amazon mollies at the beginning and end of the experiment. Body sizes were approximated from the size of detected blobs during tracking with Ethovision. Thus, body size approximations are smaller than actual fish sizes. There was no difference in body size between individuals from the control vs. predator treatment at the beginning and at the end of the experiment, but individuals in both treatments grew over time. Depicted boxplots show raw data (points), medians (horizontal lines), 25 and 75% quantiles (boxes) and 1.5 x interquartile ranges (whiskers). P-values were calculated from a pairwise post-hoc comparison of the full model (including the interaction term, see Table S6) via estimated marginal means and Tukey adjustment).
